# Tuberculosis Diagnostics in the New Millennium: Role in TB Identification and Control

**DOI:** 10.1155/2012/768603

**Published:** 2012-12-18

**Authors:** Soumitesh Chakravorty, Catharina Boehme, Jongseok Lee

**Affiliations:** ^1^Division of Infectious Diseases, Department of Medicine, New Jersey Medical School, University of Medicine & Dentistry of New Jersey, Newark, NJ 07103, USA; ^2^Foundation for Innovative New Diagnostics (FIND), Avenue de Budé 16, 1202 Geneva, Switzerland; ^3^Department of Microbiology, International Tuberculosis Research Center, Gyeongsang, Changwon 631-710, Republic of Korea

Tuberculosis (TB) continues to remain a significant threat even as we have moved into the second decade of the 21st century. As a matter of fact TB has assumed an even more ominous stance with the emergence of totally drug resistant or TDR *Mycobacterium tuberculosis* (*M. tb*) strains which are virtually untreatable. Unfortunately, there has been no new effective vaccine against tuberculosis, and in spite of introduction of several new drugs like bedaquiline, delamanid, PA824, or SQ109 and a new generation of fluoroquinolones like gatifloxacin and moxifloxacin, they are yet to be involved in the current routine antituberculosis regimen, though clinical trials for combinatorial therapy along with currently used drugs are underway. The emergence of resistance against these new classes of drugs is also a likely possibility which will result in the same problems in the future with newer group of drug-resistant strains, as we are facing today with the multidrug resistant (MDR) and extensively drug-resistant (XDR) strains. Under these circumstances, rapid and definitive molecular diagnostics for effective intervention and treatment of TB patients is a cornerstone for appropriate disease control and eradication. Recently, a lot of attention has been devoted towards rapid TB diagnostics especially those which enable rapid drug susceptibility testing (DST) to break free from the century long dependence on smear microscopy and culture methods, which are frustratingly insensitive and time consuming, respectively. WHO has recently recommended automated liquid culture systems, line probe assays, and the Xpert MTB/RIF tests which allow faster DST results and highly sensitive detection of *M. tb* from clinical samples. These assays signal towards the emerging era of rapid and decisive tests employing new generation of molecular and microbiological methods. Against this exciting backdrop of the emerging trends and innovations in new TB diagnostic techniques, the special edition of Tuberculosis Research and Treatment focuses on tuberculosis diagnostics in the new millennium, role in TB identification and control, which represents a perspective on the emerging trends in the next of generation TB diagnostics. From unconventional approaches like using sputum sniffing pouched rats to the latest immunodiagnostics like interferon gamma release assays (IGRAs), a detailed overview on the current PCR-based techniques of diagnosis of TB meningitis, as well as importance of the methods of sample collection for accurate diagnosis of TB, are dealt with in this issue. One elegant study by A. Mahoney et al. examines the utility of using pouched rats for detection of tuberculosis as an adjunct to smear microscopy in resource poor countries. The paper by T. Takahashi et al. explores the current PCR-based approaches to diagnose one of the most deadly forms of extrapulmonary TB, TB meningitis, and describes a novel “wide range quantitative nested real-time PCR” assay. Two papers by E. A. Talbot et al. and S. M. Vesenbeckh et al. deal with one of the most widely used immunodiagnostics for TB, namely, IGRA and its applicability in TB diagnostics. One paper examined its utility in overnight-stored blood samples, and a second study assessed the accuracy of the recommended cut-off values for diagnosing active TB, in which both have important implications on utility and applicability of IGRA. The paper by Mpagama et al. brings forth the importance of using overnight-pooled sputum in enhancing the sensitivity and shortening the time of detection using the BACTEC MGIT system. This special edition on the current scenario of TB diagnostics will serve as a concise but comprehensive spectrum of the different approaches aimed at faster and more definitive detection of TB disease and its control.

*Soumitesh Chakravorty* *Soumitesh Chakravorty* *Catharina Boehme* *Catharina Boehme* *Jongseok Lee* *Jongseok Lee*

